# Design of a study to determine the impact of insecticide resistance on malaria vector control: a multi-country investigation

**DOI:** 10.1186/s12936-015-0782-4

**Published:** 2015-07-22

**Authors:** Immo Kleinschmidt, Abraham Peter Mnzava, Hmooda Toto Kafy, Charles Mbogo, Adam Ismail Bashir, Jude Bigoga, Alioun Adechoubou, Kamaraju Raghavendra, Tessa Bellamy Knox, Elfatih M Malik, Zinga José Nkuni, Nabie Bayoh, Eric Ochomo, Etienne Fondjo, Celestin Kouambeng, Herman Parfait Awono-Ambene, Josiane Etang, Martin Akogbeto, Rajendra Bhatt, Dipak K Swain, Teresa Kinyari, Kiambo Njagi, Lawrence Muthami, Krishanthi Subramaniam, John Bradley, Philippa West, Achile Massougbodji, Mariam Okê-Sopoh, Aurore Hounto, Khalid Elmardi, Neena Valecha, Luna Kamau, Evan Mathenge, Martin James Donnelly

**Affiliations:** MRC Tropical Epidemiology Group, Department of Infectious Disease Epidemiology, London School of Hygiene and Tropical Medicine, Keppel Street, London, WC1E 7HT UK; Federal Ministry of Health, PO Box 1204, Khartoum, Sudan; Khartoum State Malaria Control Programme, VBDC, P.O. Box 1517, Khartoum, Sudan; School of Biological Sciences, Universiti Sains Malaysia (USM), 11800 Pulau Pinang, Malaysia; KEMRI Centre for Geographic Medicine Research Coast, P.O. Box 230, Kilifi, 80108 Kenya; National Reference Unit (NRU) for Vector Control, The Biotechnology Center, University of Yaoundé I, Messa, P.O. Box 3851, Yaoundé, Cameroon; Programme National de Lutte conte le Paludisme (PNLP), Ministère de la Santé, Cotonou, Benin; Department of Health Research, National Institute of Malaria Research, (GoI), Sector 8, Dwarka, Delhi, 110 077 India; Global Malaria Programme, World Health Organization, Avenue Appia, Geneva, Switzerland; KEMRI/CDC Research and Public Health Collaboration, PO Box 1578, Kisumu, 40100 Kenya; National Malaria Control Programme, Ministry of Public Health, PO Box 14386, Yaoundé, Cameroon; Organisation de Coordination pour la Lutte contre les Endémies en Afrique Centrale (OCEAC), Yaoundé, Cameroon; Department of Vector Biology, Liverpool School of Tropical Medicine, Pembroke Place, Liverpool, L3 5QA UK; Ministry of Health, Wad Madani, Gezera State Sudan; Malaria Programme, Wellcome Trust Sanger Institute, Hinxton, Cambridge, UK; Department of Medical Physiology, School of Medicine, College of Health Sciences, University of Nairobi, Nairobi, Kenya; Ministry of Health, Malaria Control Unit, PO Box 1992, Nairobi, 00202 Kenya; KEMRI Centre for Public Health Research, Nairobi, Kenya; Centre de Recherche Entomologique de Cotonou, Cotonou, Benin; KEMRI-Centre for Biotechnology and Research Development, Nairobi, Kenya; KEMRI-Eastern and Southern Africa Centre of International Parasite Control, Nairobi, Kenya; Faculty of Medicine and Pharmaceutical Sciences, University of Douala, PO Box 2701, Douala, Cameroon; Faculté des Sciences de la Santé, Université d’Abomey Calavi, Cotonou, Benin; School of Public Health, University of the Witwatersrand, Johannesburg, South Africa

## Abstract

**Background:**

Progress in reducing the malaria disease burden through the substantial scale up of insecticide-based vector control in recent years could be reversed by the widespread emergence of insecticide resistance. The impact of insecticide resistance on the protective effectiveness of insecticide-treated nets (ITN) and indoor residual spraying (IRS) is not known. A multi-country study was undertaken in Sudan, Kenya, India, Cameroon and Benin to quantify the potential loss of epidemiological effectiveness of ITNs and IRS due to decreased susceptibility of malaria vectors to insecticides. The design of the study is described in this paper.

**Methods:**

Malaria disease incidence rates by active case detection in cohorts of children, and indicators of insecticide resistance in local vectors were monitored in each of approximately 300 separate locations (clusters) with high coverage of malaria vector control over multiple malaria seasons. Phenotypic and genotypic resistance was assessed annually. In two countries, Sudan and India, clusters were randomly assigned to receive universal coverage of ITNs only, or universal coverage of ITNs combined with high coverage of IRS. Association between malaria incidence and insecticide resistance, and protective effectiveness of vector control methods and insecticide resistance were estimated, respectively.

**Results:**

Cohorts have been set up in all five countries, and phenotypic resistance data have been collected in all clusters. In Sudan, Kenya, Cameroon and Benin data collection is due to be completed in 2015. In India data collection will be completed in 2016.

**Discussion:**

The paper discusses challenges faced in the design and execution of the study, the analysis plan, the strengths and weaknesses, and the possible alternatives to the chosen study design.

## Background

Reductions in malaria disease burden, as documented in recent World Malaria Reports [1, 2], have coincided with the massive scale-up of malaria prevention measures, of which vector control was the predominant component, particularly in sub-Saharan Africa. The core malaria vector control interventions are insecticide-treated nets (ITNs) and indoor residual spraying (IRS), both of which deploy insecticides to kill malaria-transmitting mosquitoes. In populations at risk of malaria in sub-Saharan Africa, the proportion of households owning at least one ITN increased from 3% in 2000 to 67% in 2013, with the proportion of the population sleeping under a net increasing from 2 to 44% over the same period. In 2014, the number of ITNs delivered in the region was projected to reach 214 million. The proportion of the population at risk of malaria in the WHO African region who were protected by IRS, increased from 5% in 2005 to 11% in 2011, but fell to 7% in 2013, possibly in response to having to spray more expensive insecticides required for the management of insecticide resistance [3]. Globally 3.5% of populations at risk of malaria were protected by IRS in 2013.

Whilst these substantial efforts have had a major impact on malaria disease burden, the global burden of malaria is still unacceptably high. Worldwide there were an estimated 584,000 malaria deaths and 198 million malaria cases in 2013. However, malaria mortality rates fell by 47% and malaria cases per 1,000 persons at risk declined by 30% between 2000 and 2013. In Africa malaria infection prevalence in children aged 2–10 years reduced continent-wide from 26% in 2000 to 14% in 2013, whilst prevalence in countries with stable transmission fell from 35 to 18% over the same period [2]. Individual studies suggest that reductions in malaria incidence and infection prevalence have often occurred in the wake of the introduction or scale-up of vector control interventions [4–6].

These successes are now being threatened by the widespread emergence of insecticide resistance, especially in sub-Saharan Africa and India [3]. Resistance to pyrethroids, which is currently the only insecticide class used in ITNs, is now ubiquitous in major vectors of malaria on the African continent. Resistance to insecticides that belong to the other three chemical classes used for IRS is emerging in many regions where insecticides are used for vector control [7–9].

The Global Plan for Insecticide Resistance Management in Malaria Vectors [3] sets out the strategies that countries should employ to monitor and manage insecticide resistance. Whilst there is extensive evidence of resistance in *Anopheles* mosquitoes, there is little evidence of control programme failure associated directly with insecticide resistance largely because of many confounding factors. In KwaZulu-Natal, South Africa, a sharp increase in malaria cases from <5,000 to approximately 50,000 cases per year in the 1990s coincided with a switch in IRS insecticide from DDT to the pyrethroid deltamethrin [10]. Insecticide susceptibility tests showed that there was resistance to pyrethroids in *Anopheles funestus*, a vector that was previously driven to near extinction in KwaZulu-Natal [11]. A change in policy that re-introduced IRS with DDT in 2000 was followed by a rapid decline in cases which was maintained in subsequent years. This example is a powerful reminder of the damage that can be caused by failing insecticides, particularly if insecticide policy is not based on appropriate susceptibility testing. Although this example constitutes the strongest indication of malaria operational programme failure resulting from insecticide resistance, it is somewhat undermined by the simultaneous switch from sulfadoxine/pyrimethamine (SP) to artemether/lumefantrine as a first-line drug in 2001, due to documented drug resistance to SP in *Plasmodium falciparum* [12].

Another cited example of apparent malaria control failure due to insecticide resistance is Bioko Island, Equatorial Guinea, where IRS with deltamethrin was used against *Anopheles gambiae* s.s. which harboured a *kdr* mutation often associated with pyrethroid resistance [13]. However, subsequent detailed analyses of locality by locality disaggregation of available data cast doubt on the initial interpretation [5, 14]. A recent review [15] concluded that regardless of pyrethroid resistance, ITNs are superior to untreated nets in terms of mosquito mortality in semi-field hut trials and laboratory cone bio-assays.

These examples do not suggest that insecticide resistance does not pose a substantial and real threat to malaria vector control, but rather they demonstrate how difficult it is to assess the evidence of epidemiological impact of insecticide resistance when relying on historical data. To address this, a multi-country prospective study to assess the impact of insecticide resistance on the effectiveness of long lasting insecticidal nets (LLINs) and IRS was initiated in five countries, namely Benin, Cameroon, India, Kenya and Sudan, co-ordinated by the World Health Organization (WHO) and with primary funding from the Bill and Melinda Gates Foundation. This paper describes the design that was adopted for conducting this study.

The principal objectives of the study were:

1. To determine the impact of insecticide resistance in malaria vectors on the protective effectiveness of ITNs and IRS and hence on malaria disease burden;
2. To assess trends in the insecticide resistance status and underlying mechanisms in the main malaria vector species from the study areas in response to different interventions.

## Methods

**Figure 1:**
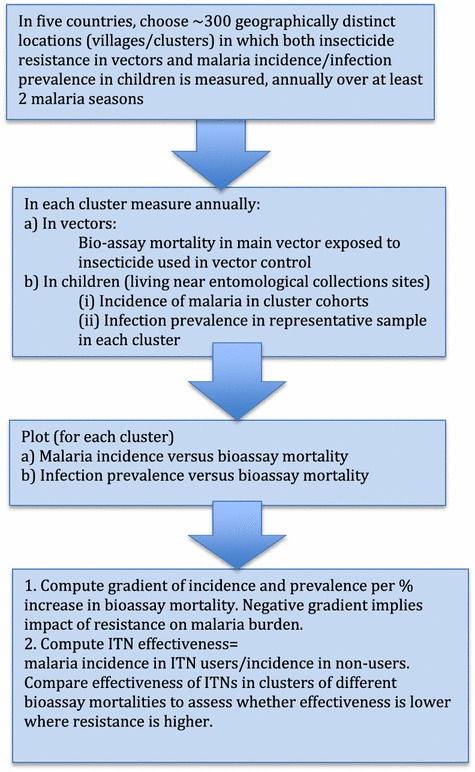
Schematic summary of the study design.

The objective of this study required an observational design since insecticide resistance (IR), the exposure of interest, could not be randomly allocated. To gain statistical power, it was necessary to conduct as many independent observations relating IR to a measure of vector control effectiveness as possible. Figure 1 summarizes the overall study design.

IR was characterized in the mosquito populations to which human populations, in whom disease burden was estimated, were exposed. Therefore IR assessments were made in mosquitoes caught in the neighbourhood of the human cohorts in which cases arose. As there was no way of knowing whether malaria cases were a result of bites from resistant or susceptible mosquitoes, information on resistance status of mosquitoes in the area was used as a proxy of individual exposure to infective IR mosquitoes. Any association between incident cases and the exposure was investigated at the level of a cluster (area). In this respect the study has an ecological study design.

In the study areas in the five countries, the impact of IR on epidemiological malaria outcomes in relation to the presence of insecticide-based interventions was assessed using one or both of the two approaches described below. The study was designed on the basis of use of LLINs at high coverage rates at all study sites in accordance with WHO policy [16].

### Approach 1

The following explanation of the study design is in terms of ITNs as a vector control intervention since ITNs were used in all study sites. A similar elaboration is implied for IRS.

**Figure 2:**
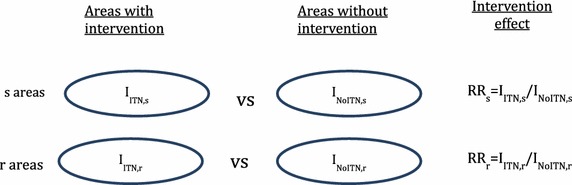
Schematic of measurement of ITN effectiveness in areas with susceptible and areas with resistant vectors.

The study outcome was vector control intervention effectiveness in localities with vectors with IR in comparison to those with susceptible vectors. Consider a group of villages randomly assigned to receive ITNs (the intervention group) and a similar group of villages assigned to not receive the intervention (the control group). Malaria incidence in the intervention group is denoted as I_ITN_ and malaria incidence in the control group is denoted as I_NoITN_. A measure of intervention effectiveness is the incidence rate ratio I_ITN_/I_NoITN_: the smaller the ratio, the more effective the intervention, other factors being similar. Effectiveness of ITNs could then be measured in areas with susceptible vectors (s areas) and in areas with resistant vectors (r areas) as illustrated in Figure 2.

Loss of effectiveness can be quantified by comparing the effectiveness of ITNs in areas with resistant vectors to the effectiveness of ITNs in areas with susceptible vectors by taking the ratio of rate ratios (RR), (I_ITN,r_/I_NoITN,r_)/(I_ITN,s_/I_NoITN,s_) where the subscripts r and s refer to resistant and susceptible areas, respectively. This ratio ranges from 0 to 1, where the smaller the ratio the larger the loss of effectiveness in areas of resistance (with a value of 1 indicating no loss of effectiveness).

Ethically, a trial with a neutral control group that was not to receive a prevention measure (i.e., vector control) was unacceptable since it is known that these interventions are effective. This was also not a viable approach given that almost all communities in malaria-endemic countries now have a certain level of ITN coverage anyway. The two quantities I_NoITN,r_ and I_NoITN,s_ therefore cannot be measured.

However, it is reasonable to assume that IR will not cause an increase in malaria incidence in places where there is no insecticide-based vector control, if all other transmission factors are comparable. If susceptible vectors are more competent than resistant vectors, then I_NoITN,r_ ≤ I_NoITN,s_. The two effects of resistance and vector competence in resistant vectors cannot be disentangled in this study; from the perspective of malaria vector control the combined impact of resistance and potentially reduced vector competence is of key importance; it is this combined impact that this study is designed to estimate.

Impact of resistance on relative risk due to the intervention

$$= \frac{{RR_{r} }}{{RR_{s} }} = \frac{{I_{ITN,r}}/{I_{{{\text{NoITN}},r}}}}{{I_{ITN,s} }/I_{{{\text{NoITN}},s}}} = \frac{{I_{ITN,r} }}{{I_{ITN,s} }}$$

if I_NoITN,r_ = I_NoITN,s_.

The outcome measure for estimating loss of effectiveness would therefore be the ratio I_ITN,r_/I_ITN,s_ where I_ITN,r_ and I_ITN,s_ refer to mean incidence in communities with ITNs where vectors are resistant and similar communities where vectors are susceptible, respectively. It is possible that areas with resistance and those without resistance may differ not just in their resistance status, but by other factors that are associated with malaria incidence. This would result in the two quantities that cannot be measured I_NoITN,r_ and I_NoITN,s_ being unequal. As far as is possible estimates of the impact of IR should be adjusted for such confounders if they are measurable.

**Figure 3:**
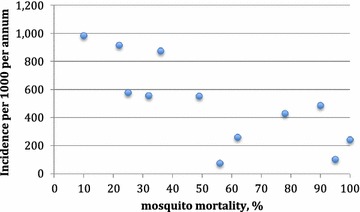
Hypothetical data of cluster specific malaria incidence in relation to hypothetical cluster specific mosquito mortality from WHO bioassay susceptibility tests.

IR was measured annually as it was likely to change during the course of the study. It was therefore treated as a time-dependent exposure in the analysis. The hypothesis of higher average malaria incidence in localities of lower susceptibility to insecticide is illustrated with hypothetical data in the scatterplot of cluster-specific malaria incidence versus cluster-specific mosquito mortality in Figure 3.

Even though no randomization of the exposure (IR) was possible, a cluster design was used to generate as large a number of observations of malaria outcomes and IR as feasible, and thereby gain statistical power. Each cluster acted as an entomological and as an epidemiological sampling point. Clusters were chosen to be villages or groups of hamlets with at least 500 houses that were at least 2 km apart but with greater separation where possible [17]. Households for the assessment of epidemiological outcomes were selected on the basis of close proximity to the habitats at which mosquitoes were collected for measurement of IR. In Sudan and India, clusters could be randomly allocated to receive either IRS in combination with ITNs, or ITNs alone. These two country studies had the advantage of allowing the assessment of the differential impact of resistance on the combination of interventions relative to a single intervention, and the evaluation of differences in temporal trends in resistance due to differences in selection pressure exerted by the combination relative to the single intervention.

### Approach 2

ITN effectiveness can also be assessed by comparing infection prevalence between ITN users and non-users in cross-sectional surveys. This is in principle a case control study design comparing ITN usage between infected (case) and non-infected (control) individuals. In a cross-sectional survey the protection offered by ITNs can be estimated in each cluster from the prevalence odds ratio

$${\text{OR}}_{{{\text{ITN}}\;{\text{versus}}\;{\text{NoITN}}}} = \left[ {{\text{P}}_{\text{ITN}} /\left( { 1- {\text{P}}_{\text{ITN}} } \right)} \right]/\left[ {{\text{P}}_{\text{NoITN}} /\left( { 1- {\text{P}}_{\text{NoITN}} } \right)} \right]$$

where P_ITN_ is the infection prevalence in ITN users and P_NoITN_ is the infection prevalence among non users of nets. If the ITNs are effective, one would expect OR_ITN versus NoITN_ <1; if they are less effective or ineffective one would expect this odds ratio to be close to 1. Estimating infection prevalence in clusters with resistant and susceptible vectors would yield a measure of the impact of IR by computing the ratio of odds ratios

$${\text{OR}}_{{{\text{ITN}}\;{\text{versus}}\;{\text{NoITN}},{\text{ r}}}} /{\text{OR}}_{{{\text{ITN}}\;{\text{versus}}\;{\text{NoITN}},{\text{ s}}}}$$

for all clusters combined. Alternatively, retaining resistance as a continuous variable, the loss of effectiveness of ITNs could be assessed by estimating the coefficient of the linear regression of OR_ITN versus NoITN_ on mosquito susceptibility, i.e., computing the slope of the plot of OR_ITN versus NoITN_ versus mortality.

### Primary outcomes

In all five countries, malaria incidence was measured by active case detection in cohorts of children recruited in each study cluster. A total of 44,720 children were followed up. Prevalence of infection was measured through cross-sectional surveys, also in each cluster, in Sudan, Kenya, Cameroon and Benin. Active infection detection was carried out in the three high transmission study areas (Benin, Cameroon and Kenya), again in cluster specific cohorts. Although passive case detection data were available in Benin and India, the reliability of these data could not be guaranteed and this indicator was therefore not intended for primary analysis. Each of the outcome indicators were measured in each study cluster. Table 1 shows which outcomes were measured in each of the five countries.

**Table 1:** Epidemiological outcomes, by country

Epidemiological outcome indicator	Clinical malaria incidence (fever plus infection)	Infection incidence	Infection prevalence	Malaria case incidence by passive case detection^a^
Method	Active case detection by testing cohort children who are febrile or report recent fever	Active infection detection by testing cohort children at 2 weeks intervals	Malaria indicator surveys testing a randomly selected cross-section of children in study clusters	Passive case detection using clinic registers based on confirmed cases
Benin	X	X	X	X
Cameroon	X	X	X	
India	X			X
Kenya	X	X	X	
Sudan	X		X	

^a^Passive case detection will only be used as supplementary data.

The four epidemiological outcome indicators are described below.

#### Clinical malaria incidence from active case detection cohorts

Cohorts of children were recruited in each study cluster (see Table 2 for size of cohorts in each country) in close proximity to the site from which mosquitoes were collected to test for susceptibility to insecticide. Children were enrolled into the cohort if the parent or caregiver consented after receiving an explanation of the study procedures. Cohort members were visited weekly or fortnightly by a community health worker during the malaria season. If a child was febrile, or had a recent history of fever, they were tested for malaria infection by RDT. For the purpose of estimating malaria incidence, a malaria case diagnosis was defined as having a positive blood test and having fever or having had recent fever. The community health worker would also ask whether the child had visited a health facility since they were last seen and investigate whether a blood test was done that confirmed the diagnosis of malaria. Children who tested positive were treated according to national treatment guidelines, or were referred to a local health facility for treatment. For a period of 2 weeks following treatment, children in cohorts were regarded as being under prophylaxis and hence not at risk. Use of ITNs the night before the visit was assessed by interviewing the household head or caregiver at each visit. The condition of nets was observed and recorded periodically. Active case detection cohorts were followed continuously, but children were released from cohorts when they had reached the upper age limit, upon which they were replaced by a younger child.

**Table 2:** Study implementation details by country

	Sudan	Kenya	Cameroon	Benin	India
Outcome indicators					
Active case detection: average cohort size per cluster; age group in years	200; 0.5–10	80; 0.5–5	80; 0.5–5	70; 0.5–5	93; 0.5–14
Active infection detection: cohort size per cluster; age group in years		20; 0.5–5	20; 0.5–5	30; 0.5–5	
Cross sectional prevalence of infection (✔); sample per cluster; age range in years	✔; 100; 0.5–10	✔; 50; 0.5–5	✔; 80; 0.5–5	✔; 40; 0.5–5	
Passive case detection from clinic registers (✔)				✔	✔
Statistical power assumptions					
Number of clusters	140 (66 sentinel, randomly selected by study arm)	50	38	32	80
Indicator	Active case detection incidence	Active case detection incidence	Active case detection incidence	Active case detection incidence	Active case detection incidence
Power; significance, k^a^	80%; 5%; 0.5	80%; 5%; 0.4	80%; 5%; 0.4	80%; 5%; 0.4	80%; 5%; 0.3
Minimum detectable difference in incidence between low and high resistance clusters/rate ratio high to low resistance	30%; 1.3	40%; 1.4	50%; 1.5	54%; 1.54	50%; 1.5
Assumed incidence in low resistance clusters; number of years follow-up	0.030 per annum; 3	1.4 per annum; 2	0.6 per annum; 2	1.4 per annum; 2	0.015 per annum; 2
Study schedule	2011–2015	2012–2015	2012–2015	2012–2015	2013–2016

^a^Coefficient of variation in incidence between clusters [18].

#### Infection incidence in cohorts with repeated testing regardless of symptoms

In the three high-transmission countries, cohorts of children under 5 years of age were recruited in each cluster and visited fortnightly (see Table 2). At recruitment and following written consent, all children were given a treatment dose of the first-line anti-malarial drug for the particular country [either artemether/lumefantrine (AL) or artesunate/amodiaquine (ASAQ)], for clearance of any parasites. At the first visit following treatment (visit 1), a blood-slide was taken for microscopic examination to verify that children had been successfully cleared of parasites; those positive were treated or referred for treatment, and released from the cohort. The period between recruitment and visit 1 was not included as time at risk for the purpose of incidence calculations since children were assumed to be protected by the prophylactic effect of treatment. At subsequent visits, children were tested by RDT. In Cameroon and initially in Kenya only those having symptoms (febrile) were tested every second fortnightly visit whilst all children were tested at least monthly. Details of visits to health facilities which took place between scheduled health worker visits, were recorded. Once a child tested positive they were treated and left the cohort. A new active infection detection (AID) cohort was recruited each year at the start of the malaria season. Whether the child slept under an ITN the night before the visit was determined at each visit from the caregiver. The physical condition of nets was observed and recorded at the time of recruitment.

#### Prevalence of infection with *Plasmodium* parasites

Cross-sectional malaria indicator surveys were performed in each study cluster to determine prevalence of infection of malarial parasites in children. The frequency of surveys and the age range of children eligible for inclusion varied between countries. Generally, blood tests were performed by RDT; in Benin microscopic examination of blood films was carried out. Survey questionnaires were adapted from the standard RBM Monitoring and Evaluation Reference Group malaria indicator survey and included questions on ITN use [19]. In Sudan, dried blood spots on filter paper were collected for PCR analysis on a subsample of specimens. For molecular analysis, *P. falciparum*, was detected using real-time PCR assays from blood spots [20].

#### Clinical malaria case incidence from passive case detection

Incidence of malaria could be determined by passive case detection if a reliable system for reporting outpatient cases was in place, if a policy of testing all children under five presenting with a fever for malarial parasites was adhered to, if no stock-outs of slides or rapid diagnostic tests (RDTs) occurred and if diagnosis of malaria was only made if the patient tested positive by RDT or microscopic blood-slide examination. In addition, the place of residence of reported cases had to be accurate enough to determine which study cluster the patient resided in. Passive case detection data were collected in Benin and India only, where these conditions were partially met. Passive case detection data were not used as a primary outcome indicator since their quality could not be guaranteed. Passive case detection served to supplement the more rigorously derived indicators.

### Exposure variables

**Figure 4:**
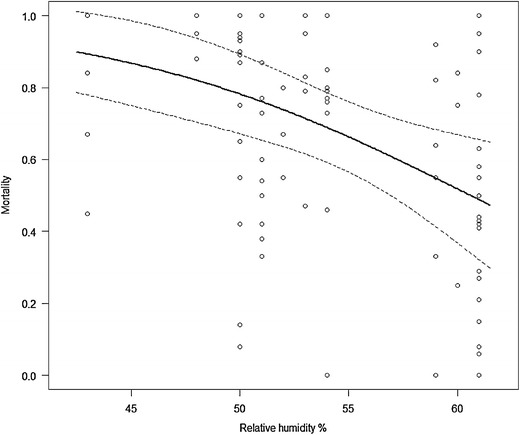
The relationship between insecticide mortality and relative humidity in isofemale collections of *Anopheles gambiae* from Tororo, Uganda exposed to 0.75% permethrin for a population specific LT50 (the exposure time required to kill 50% of the population after a 24 h holding period). The *line * in *bold* is a logistic regression of Mortality on relative humidity at time of exposure. (Mortality ~7.30 −0.12 RH; likelihood ratio test p < 0.0001; pseudo R^2^ = 0.163) (Muller et al. unpublished).

The primary exposure variable, measured annually in each study cluster, was phenotypic susceptibility status to pyrethroids of the predominant local vector/s, measured as per cent mortality according to WHO adult susceptibility tests. In the majority of cases these adults were reared in the insectary from larvae collected in breeding sites within each study cluster. In certain cases (e.g. India) where larval sites were not detected, resting females were used, in line with WHO recommendations [21]. Female *Anopheles* mosquitoes were exposed to insecticides using WHO impregnated papers for 60 min at standard concentrations [21]. The mosquitoes were then maintained, where possible, at a temperature of 25 ± 2°C and humidity of 80 ± 5%; mortality was measured at 24 h post-exposure. Inadequate temperature and humidity control can result in excess mortality, illustrated in Figure 4. There is increasing evidence that WHO resistance prevalence assays may be an insensitive means of measuring changes in resistance, particularly when the level/intensity of resistance is high [22] and there are a number of novel approaches including modified CDC bottle bioassays and time/dose response assays that may be more sensitive [23–25]. However, the power of this study was reliant upon obtaining a quantitative estimate of resistance for each study cluster and we took the pragmatic decision to maximize the number of clusters for which we obtained phenotypes at the expense of arguably more sensitive, but more laborious, intensity assays.

In areas in which the insecticide in most ITNs was deltamethrin (for example PermaNet^®^ 2.0), susceptibility tests were conducted with deltamethrin. In areas where ITNs were in use in which permethrin was the active ingredient (for example, Olyset^®^ net), susceptibility tests were carried out using this insecticide.

Allelic frequency of the *Vgsc*-*L104F* (formerly *kdr*-*west*) and *Vgsc*-*L1014S* (formerly *kdr*-*east)* mutation in *Anopheles arabiensis* and/or *An. gambiae* s.s. was determined in samples of locally caught mosquitoes to provide a second measure of IR [26, 27]. The extent to which these mutations are associated with phenotypic resistance is variable and possibly location dependent [28, 29]. Resistance, whether phenotypic or genotypic, was observed as a continuous measure on a percentage scale.

### Sample size considerations

For each country, a power calculation was performed to estimate a minimum difference in incidence or prevalence that could be detected between high and low resistance clusters, based on the total number of clusters that could be formed with reasonable separation distance between clusters, and in which separate assessments of IR could be made. For the purpose of sample size calculations it was assumed that there would be an equal number of low and high resistance clusters, i.e., that the clusters would be dichotomized based on median mosquito mortality. The total numbers of clusters for each country were as follows: Sudan (140), Kenya (50), Cameroon (38), Benin (32), India (80). Details for each country are given in Table 2.

### Data management

Data capture and management systems were set up in each country by information technology specialists. The structure of databases was standardized across countries. In case detection cohorts, follow-up was censored (excluded) if visits were missed, but children “re-entered” follow-up if they were observed at subsequent visits. Missing resistance data for a cluster meant that the cluster could not be included in analysis for the particular year.

### Ethics approval

In each country ethics approval for the study was obtained from the relevant national research ethics committee. Participation in the study was voluntary and based on written informed consent by the participant, or the caregiver in the case of children.

### Country implementation

Malaria endemicity and baseline IR for the study settings are shown in Table 3. Key implementation details are shown in Table 2.

**Table 3:** Study settings by country

	Sudan	Kenya	Cameroon	Benin	India
Study locations	El Hoosh and Hag Abdalla (Gezira State); Galabat (Gedarif State); New Halfa (Kassala State)	Districts of Teso, Rachuonyo, Nyando and Bondo (Western Kenya)	Districts of Garoua, Pitoa and Mayo Oulo (North Region)	Districts of Ifangni, Sakété, Pobé and Kétou (Departement de Plateau)	Subdistrict of Keshkal (Kondagaon, Chhattisgarh)
Predominant malaria vectors	*An. arabiensis*	*An. gambiae* s.s.*, An. arabiensis* and *An funestus*	*An arabiensis, An. gambiae* s.s. and *An funestus*	*An. gambiae* s.s.	*An. culicifacies*
Vector control Interventions	High coverage of LLINs (PermaNet 2.0) in all study clusters. In each study area half of clusters randomly allocated to receive additionally IRS with bendiocarb^b^, balanced by baseline *kdr* frequencies	High coverage of LLINs (PermaNet 2.0 and Olyset Net) in all clusters. Rachuonyo and Nyando received IRS with deltamethrin and lambda-cyhalothrin in 2012, but no IRS was carried out subsequently	High coverage of LLINs (PermaNet 2.0) in all clusters	High coverage of LLINs (primarily Olyset Net) in all clusters	High coverage of LLINs (PermaNet 2.0) in all clusters. Half of clusters randomly allocated to receive additionally IRS with bendiocarb
Baseline insecticide resistance (cluster-specific range)	Kdr frequency by cluster ranged from 8.3 to 70.8% (2010); WHO Bioassay mortality to deltamethrin in sentinel clusters ranged from 47 to 100% (2011)	Kdr frequency by cluster not available at baseline (2011); WHO Bioassay mortality to deltamethrin ranged from 1 to 100% (2011)	Kdr frequency by cluster ranged from 9 to 65% (2011) WHO Bioassay mortality to deltamethrin ranged from 43 to 100% (2012)	Kdr frequency by cluster ranged from 44 to 93% (2011) WHO Bioassay mortality to deltamethrin ranged from 20 to 100% (2011)	WHO Bioassay mortality to deltamethrin ranged from 86 to 100%; WHO Bioassay mortality to bendiocarb ranged from ranged from 27 to 98%
*PfPR*_*2*–*10*_ Endemcity class^a^	Low	High	High	High	Low

^a^ *PfPR*_*2*–*10*_ is the proportion of 2–10 year olds in the general population that are infected with *P. falciparum*, averaged over the 12 months of 2010 as estimated by Malaria Atlas Project (MAP) [30]; low = 0% < *PfPR*_*2*–*10*_ ≤ 5%; intermediate = 5% < *PfPR*_*2*–*10*_ ≤ 40%; high = *PfPR*_*2*–*10*_ > 40%.

^b^In Galabat deltamethrin was sprayed in 2011 and 2012.

In Sudan and India clusters were randomized to receive either universal coverage with ITNs alone or IRS plus universal coverage of ITNs in combination. This allowed an additional evaluation to be conducted, the comparison of the combined use of IRS and ITNs with ITNs alone, in varying resistance settings. In Sudan, this randomization was restricted in such a way that the baseline frequency of genotypic IR in local vector populations was approximately equal in the two study arms, based on molecular analysis of local mosquitoes caught in each cluster at baseline [31]. In India, randomization was carried out separately in strata defined by the median of standard bioassay mortality of *Anopheles culicifacies* to bendiocarb, carried out during baseline entomological surveys in each study cluster.

### Analysis plan

All variables (outcome, exposure and confounders) were measured in each study cluster at different time points and analysis took account of the time dependent nature of the measurements. Although separate analyses are planned for each country, the statistical methodology described below is consistent across all countries.

For infection incidence (infection detection cohorts) and case incidence (case detection cohorts), incidence was estimated as the number of incident cases per child year of follow-up. Incidence RRs (or hazard ratios where appropriate) were calculated per 1% change in cluster specific vector susceptibility (mosquito mortality) or as incidence RRs in high resistance clusters, relative to low resistance clusters. Multiple variable Poisson or Cox regression was used to adjust estimated effects for measured potential confounders where appropriate. For cross-sectional prevalence of infection data, logistic regression was used to estimate odds ratios, again per % change in cluster specific mosquito mortality or comparing prevalence in high versus low resistance clusters. All estimates of epidemiological outcomes used statistical methods that took account of the variation between clusters by either using random effects models, or the first-order Taylor-series linearization method as a robust variance estimator to calculate appropriate standard errors [32, 33].

When resistance was analysed as a dichotomous variable (‘high’ versus ‘low’ resistance), the median phenotypic and/or genotypic resistance value for all clusters was used as the cut-point, with each cluster classified as low or high resistance depending on which side of the cut-point it fell. Fixed definitions such as the WHO definition of susceptibility [3] of a minimum threshold of 98% mortality in bioassay tests were not used, since there were generally too few or no clusters with susceptibility according to this definition. Moreover, such predefined thresholds do not necessarily reflect operational failure. Whether the relationship between case incidence and resistance was linear could not be pre-defined; therefore analyses using resistance as categorical and continuous variables were carried out.

In countries where clusters were randomized to LLINs alone versus LLINs plus IRS (Sudan and India), the effect of the combination compared to one method alone was assessed; the effect of resistance on the effect of the combination was estimated as an effect modifier (interaction) in a multiple variable model. Table 4 is an illustration, using hypothetical data, of the way the results would be presented for clusters that were randomized to a single intervention (LLINs alone) versus combined interventions (LLIN plus IRS), illustrating how loss of effectiveness of the second intervention can be expressed as a ratio.

**Table 4:** Tabulation of hypothetical data on incidence by active case detection in high and low resistance clusters, showing loss of effectiveness of second intervention (IRS)

Insecticide resistance	Vector control	Incidence (cases per 1,000 person years)	Rate ratio (LLIN + IRS versus LLIN)	Change in effectiveness ratio (high versus low resistance)
Low	LLIN	100	1	
	LLIN + IRS	40	0.4	1
High	LLIN	100	1	
	LLIN + IRS	80	0.8	2.0 [95% CI 1.1–5.0; p = 0.04]

For Approach 2, effectiveness of LLINs was calculated as an odds ratio for infection prevalence between net users and non-users, in high and low resistance clusters, respectively. Effectiveness odds ratios were compared through an interaction term in the model as a ratio of the two odds ratios. A similar analysis was carried out using incidence rate ratios for comparing incidence rates in users and non-users of ITNs.

Table 5 illustrates with hypothetical data how the estimated of loss of effectiveness of nets due to resistance was estimated as an interaction, expressed as a ratio of odds ratios (>1 showing loss of effectiveness).

**Table 5:** Hypothetical illustration of infection prevalence by ITN use and by resistance stratum demonstrating loss of effectiveness expressed as an odds ratio

Deltameth. resistance (2012) of clusters (no. of clusters)	ITN-use	Infection prevalence, % (N)	ITN effectiveness	Effect modification of resistance on effectiveness
			Odds ratio ITN-use versus no net use	Change in effectiveness ratio, (high versus low resistance)
Low resistance (mortality ≥80%)	No	49	1	
	Yes	27	0.4	1
High resistance (mortality <80%)	No	37	1	
	Yes	30	0.7	1.75 [1.1–5.0]

## Discussion

Previous research on IR has generally been restricted to entomological outcomes. As a consequence there is little guidance on what the magnitude of its effect on malaria incidence is likely to be. Quantifying the health impact of IR is challenging, as no random allocation of the exposure is possible. A possible alternative design would have been to randomly allocate treated and untreated nets to populations in areas of known IR to see whether the treated nets still conferred additional protection compared to untreated nets. This would be based on the assumption that ITNs in the presence of resistance are, at worst, equivalent to untreated nets. A recent meta-analysis of entomological studies on pyrethroid resistance [15] concluded that ITNs are more effective against malaria than untreated nets, regardless of resistance. It would therefore be unethical to randomize individuals to receive either insecticide-treated vector control tools (ITNs) or untreated nets.

It is not known whether resistant mosquitoes are as competent as susceptible mosquitoes as vectors of malaria. If resistant mosquitoes are less efficient vectors, as seen in a study of filariasis transmission [34], this effect could partially offset any loss of effectiveness of vector control due to IR. This study will only be able to measure the overall impact of IR in malaria vectors on vector control, without disentangling the separate effects of increased survival and possible loss of vector competence.

The results of an observational study may be subject to confounding. A way of guarding against this is through adjustment in the analysis for known confounders although this does not provide a guarantee against unknown and unobserved confounders. Nevertheless, well-designed, non-randomized, observational, epidemiological studies using long-term follow-up of cohorts have convincingly shown the effects of exposures that are harmful to human health, e.g., smoking [35], as well as exposures that are beneficial, e.g., physical exercise [36]. When such studies are ‘ecological’ studies because they rely on associations at community rather than individual level, an additional layer of complexity is involved. The association between the outcome (e.g., malaria incidence/prevalence) and an exposure (e.g. IR) at area level is sometimes referred to as ecological inference [37] and can be subject to hidden confounding unless the association is also true at individual level. In this case there was no means of verifying an association between individual incident malaria cases and individual resistant mosquitoes.

Further challenges were the collection of malaria incidence data in countries where reporting systems are weak, and difficulties collecting sufficient mosquito larvae from each cluster for susceptibility testing. It was necessary to set up active case detection cohorts for the measurement of incidence, which is logistically challenging and requires intense supervision. Measuring IR in many locations is also challenging and requires standardization of procedures and testing conditions such as temperature and humidity. There are more sensitive methods of resistance determination such as generation of population specific concentration or lethal exposure time curves [24, 38]. However these better metrics of the intensity of IR require multiple exposures per cluster which was not possible in logistically constrained study settings. The WHO susceptibility test measuring mosquito mortality within 24 h after 60 min of exposure to a standard concentration of insecticide on impregnated papers is commonly used and was considered operationally the most feasible.

The second approach described above, in which loss of effectiveness is measured as the ratio of odds ratios (interaction) in users and non-users of nets in high and low resistance clusters, respectively, has the advantage that the non-ITN users serve as a reference group. The drawback is that ITN use was not randomly allocated to individuals and hence there are major confounders affecting the relationship between infection prevalence and ITN use. Very often ITN use is greatest amongst people with highest risk. For example, according to the 2014 World Malaria Report [2] pregnant women and children were more likely than the general population to sleep under an ITN since net distribution campaigns often target these groups. This in turn may lead to apparently higher malaria prevalence overall amongst ITN users than non-users. As a result it will appear as if ITNs are ineffective. An additional problem with this approach is that reported net use (whether accurate or not) is often very high in cohorts where the standard question of net-use last night is asked at every visit. As a result, the non-user comparison group is very small and probably unrepresentative, leading to a biased estimate.

Despite these challenges, this study will generate approximately 300 observations of malaria incidence in communities protected by LLINs (in some instances in combination with IRS), and concomitant IR across multiple time points and transmission settings. If IR has a profound impact on the effectiveness of these interventions, an association between these two measures should be evident. The advantage of the chosen design is that the formation of many study clusters results in a relatively large sample of independent paired observations of IR and malaria incidence. The harmonization of methods across countries will provide the possibility of combining data across countries for analysis.

The historical data from South Africa [10] suggest as much as a ten-fold increase in malaria incidence if there is resistance to the IRS insecticide. On the other hand, studies comparing the effectiveness of ITNs and untreated nets [39] showed that untreated nets are approximately half as effective as treated nets in settings of insecticide susceptibility, suggesting a potential two-fold increase in malaria burden resulting from resistance to insecticides used on ITNs, other factors remaining the same. This study will be able provide some evidence based insights into this question.

## Conclusion

The multi-country study described in this paper, despite many inherent challenges associated with study design and measurement of resistance, is unique in assembling a large data set of connected entomological and epidemiological observations. It will provide quantitative insights on the impact that IR has on the effectiveness of malaria vector control, and the potential for control programme failure. The findings will be important for determining the urgency with which management of IR should be undertaken, and the effort and investment that are required in the search for alternatives to current insecticide-based tools for the control and elimination of malaria.
